# The influence of ketamine’s repeated treatment on brain topology does not suggest an antidepressant efficacy

**DOI:** 10.1038/s41398-020-0727-8

**Published:** 2020-02-04

**Authors:** Natalia Gass, Robert Becker, Jonathan Reinwald, Alejandro Cosa-Linan, Markus Sack, Wolfgang Weber-Fahr, Barbara Vollmayr, Alexander Sartorius

**Affiliations:** 1 Research Group Translational Imaging, Department of Neuroimaging, Central Institute of Mental Health, Medical Faculty Mannheim, Heidelberg University, Mannheim, Germany; 2 Department of Psychiatry and Psychotherapy, Central Institute of Mental Health, Medical Faculty Mannheim, Heidelberg University, Mannheim, Germany; 3 Research Group In Silico Pharmacology, Central Institute of Mental Health, Medical Faculty Mannheim, Heidelberg University, Mannheim, Germany; 4 Research Group Animal Models in Psychiatry, Department of Psychiatry and Psychotherapy, Central Institute of Mental Health, Medical Faculty Mannheim, Heidelberg University, Mannheim, Germany

**Keywords:** Depression, Molecular neuroscience

## Abstract

As ketamine is increasingly used as an effective antidepressant with rapid action, sustaining its short-lived efficacy over a longer period of time using a schedule of repeated injections appears as an option. An open question is whether repeated and single administrations would affect convergent neurocircuits. We used a combination of one of the most robust animal models of depression with high-field neuroimaging to perform a whole-brain delineation of functional mechanisms underlying ketamine’s effects. Rats from two genetic strains, depressive-like and resilient, received seven treatments of 10 mg/kg *S*-ketamine (*N* = 14 depressive-like, *N* = 11 resilient) or placebo (*N* = 12 depressive-like, *N* = 10 resilient) and underwent resting-state functional magnetic resonance imaging. Using graph theoretical models of brain networks, we compared effects of repeated ketamine with those of single administration from a separate dataset of our previous study. Compared to single treatment, repeated ketamine evoked strain-specific brain network randomization, resembling characteristics of the depressive-like strain and patients. Several affected regions belonged to the auditory, visual, and motor circuitry, hinting at possible cumulative side effects. Finally, when compared to saline, repeated ketamine affected only a few local topological properties and had no effects on global properties. In combination with the lack of clear differences compared to placebo, our findings point toward an inefficacy of ketamine’s long-term administration on brain topology, making questionable the postulated effect of repeated administration and being consistent with the recently reported absence of repeated ketamine’s antidepressant efficacy in several placebo-controlled studies.

## Introduction

Ketamine has a rapid antidepressant action, which is, however, relatively short-lived, typically lasting 7 days^1–3^. To prevent a post-ketamine depressive relapse, several studies tested a repeated administration as a possible maintenance strategy and demonstrated sustained antidepressant efficacy at least throughout the duration of the infusion period^4–12^. In rodents repeated treatment with ketamine reverses depressive-like behavior in chronic unpredictable stress model^13,14^ and chronic mild stress models of depression^15^.

In our previous study we investigated the long-term effects of a single administration of ketamine^16^ using the negative cognitive (NC) model^17^, one of the most robust and well-validated animal models of treatment-resistant depression (TRD)^18^. It comprises breeding two genetic strains of rats, based on their susceptibility to develop stress-escape behavior, when exposed to electrical footshocks^17^. The NC strain failing to escape represents cognitive aspects of depression, in which events are considered negative and uncontrollable, and displays the pessimistic response bias in cognitive tasks^19^. The resilient strain displays escape behavior (positive cognitive (PC) state) and represents a non-depressed phenotype^17^. In this study ketamine and placebo were administered to both strains. In contrast to the PC group, the NC strain exhibited strain-specific effects of ketamine on brain topology mainly localized within the habenula-midthalamic-hippocampal circuit^16^. This neurocircuit mediates cognitive flexibility, an ability to adaptively switch behavior depending on situation or situational context^16^, profoundly impaired in depression^20,21^. We suggested that ketamine mediates its pro-cognitive effects by normalizing the disrupted functional wiring within the habenula-thalamic-hippocampal cognitive circuitry, which might be a key imaging correlate of its long-term effect.

However, whether ketamine’s repeated administration would affect convergent neurocircuits, implying neural mechanisms similar to a single treatment, remains an open question. To address this question, we compared the effects of repeated and single types of ketamine treatment on brain circuitry. Depressive-like and resilient rats from the same genetic generation, as in our previous study, received seven daily injections of ketamine or placebo (saline). Unlike most human studies on repeated ketamine lacking placebo group^4–8,10–12^, both rat strains had parallel control groups receiving saline. After conducting a series of daily ketamine/saline injections, the animals underwent resting-state functional magnetic resonance imaging (rs-fMRI). Focusing on finding the neural basis of repeated ketamine’s action in alignment with the Research Domain Criteria (RDoC) principles^22^ and to gain insights into the fundamental brain network alterations induced by this treatment, as compared with the single administration from our previous study, we used graph theoretical models of brain networks to perform a whole-brain delineation of functional mechanisms underlying ketamine effects.

## Materials and methods

### Experimental design

**Fig. 1:**
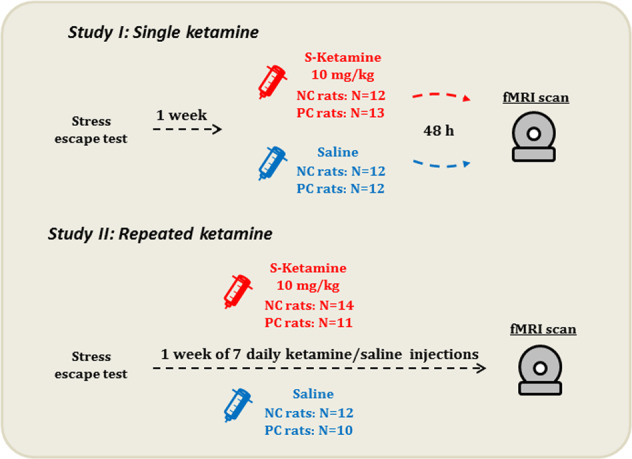
An overview of the experimental design. *NC* negative cognitive state strain, *PC* positive cognitive state strain.

Four parallel groups of the depressive-like NC and resilient PC male rats (*N* = 47; Sprague–Dawley; 83rd genetical generations; 8 weeks old; 246–344 g) underwent an escape test (for description see Supplement) and starting from this day received seven daily injections of either *S*-ketamine at 10 mg/kg (Ketanest, Pfizer Pharma GmBH, Berlin, Germany) (14 NC, 11 PC rats) or saline (12 NC, 10 PC rats) (subcutaneously (s.c.), 1 ml/kg) (Fig. 1). We chose the dose of 10 mg/kg as it corresponds to 0.5 mg/kg dose used in humans^23,24^, resulting in a similar range of peak concentrations^25^, and is an optimum antidepressant dose in the animal models^13,15^. In humans a regimen for ketamine’s repeated treatment mainly comprises six to eight intermittent injections; however, in rats a schedule is often consecutive due to faster metabolic rates in rodents^26^. Similar to an intermittent regimen, in a rat model of depression it demonstrates robust antidepressant-like effects^13^. We used a consecutive regimen in order to fit the injection schedule into a week instead of prolonging it to 2 weeks by intermittent treatments, thus keeping the ages of rats in alignment with our previous study and avoiding brain aging effects.

On the day following the last injection the animals underwent fMRI scanning. Group (PC/NC), treatment (ketamine/saline), and time of day were randomized in the fMRI measurements. During the experiments the investigator was blinded to the assignment of groups.

For the single injection, we used a separate previously acquired fMRI dataset consisting of NC and PC rats belonging to the same genetic generation as rats in the current dataset and treated with 10 mg/kg *S*-ketamine (12 NC, 13 PC rats) or saline (12 NC, 12 PC rats)^16^.

The experimental procedures (behavioral test, fMRI scanning, anesthesia protocol) and pre-processing pipeline for both datasets were identical.

Due to the exploratory nature of this study, no formal power or sample size estimation was carried out; however, the group sizes (*N* = 10–14 per group) were toward the high end of the range typically used in animal fMRI experiments.

The rats were housed in plastic cages (two rats per cage) at a constant temperature of 22 °C and 12-h light–dark cycle (lights on at 07:00 a.m.) and with food and water available *ad libitum*. At the end of the experiments the rats were sacrificed. The experiments were performed according to the regulations covering animal experimentation within the European Union (European Communities Council Directive 86/609/EEC) and within the German Animal Welfare Act, and were approved by the German animal welfare authorities (Regierungspräsidium Karlsruhe).

### MRI acquisition and pre-processing

The rs-fMRI experiments were carried out at 9.4 T MRI scanner (Bruker BioSpec, Ettlingen, Germany) with Avance III hardware, BGA12S gradient system (maximum strength 705 mT/m) and Paravision 6 software. We used a linear whole-body volume transmitter coil combined with an anatomically shaped four-channel receive-only coil array. The rats were initially anesthetized with 4% isoflurane (Baxter Deutschland GmbH, Unterschleissheim, Germany) in a mixture of 70% N_2_ and 30% O_2_; then, after positioning in the scanner, isoflurane level was reduced to 2.5% and medetomidine (Domitor, Janssen-Cilag, Neuss) was injected as a bolus (0.5 ml, 0.07 mg/kg, s.c.). Isoflurane administration was slowly discontinued during 10 min (reduction by 0.5% every 2 min), and after switching it off, the animals continuously received medetomidine at 0.28 mg/kg/h. We monitored sedation depth via recording the physiological (respiratory and cardiac) parameters throughout the experiment at 10-ms resolution using the signal breakout module (Small Animal Instruments Inc., NY, USA) and a 4-channel recorder (Velleman® N.V., Gavere, Belgium). The physiological parameters stabilized at 15 min after the start of continuous medetomidine and remained stable during the whole experiment.

The MRI acquisition protocol included a T2*-weighted echo-planar imaging (EPI) free induction decay sequence (repetition time/echo time (TR/TE) 1500/17.5 ms, flip angle 60°, field of view 35 × 35 mm^2^, voxel dimension 0.365 mm, 30 coronal slices, slice thickness 0.5 mm, 340 acquisitions over 8.5 min, 8 dummy scans), a 3D double gradient echo FieldMap sequence for correction of geometric distortions (TR = 20 ms, short TE = 1.7 ms, long TE = 5.7 ms), and a T2-weighted rapid acquisition with refocused echoes (RARE) sequence (RARE factor 16, TR/TE 1200/50 ms, flip angle 180°, the voxel dimension 0.15 × 0.15 × 0.3 mm^3^, acquisition time 23 min).

Image pre-processing was performed as in our previous studies^27,28^ and comprised the following steps: (1) correction of each EPI time-series for magnetic field (*B*0) inhomogeneities and movement using “realign & unwarp” SPM function (SPM8: http://www.fil.ion.ucl.ac.uk/spm/software/spm8); (2) regressing out the estimated movement parameter vectors from each voxel (FSL, version 4.1. http://www.fmrib.ox.ac.uk/fsl); (3) filtering out respiratory and cardiac signals using Aztec software;^29^ (4) slice-timing correction (SPM8); (5) spatial normalization to a rat brain template in Paxinos space (SPM8);^30^ (6) filtering out the cerebrospinal fluid signal from the normalized images (FSL); (7) scrubbing data frames with a frame-wise displacement >0.03 mm (linear interpolation) in order to capture the remaining motion-related artifacts;^31^ (8) global signal regression; (9) band-pass filtering (0.01–0.1 Hz) (Analysis of Functional NeuroImages version 2)^32^.

### Graph theoretical analysis

#### Graph generation

Fully connected weighted graphs covering the whole brain^33^ were created by calculating pairwise Pearson’s correlation coefficients between time-courses of 43 bilateral regions defined by Schwarz atlas^30^. The graph of each subject and measurement was normalized, that is, divided by the maximum value within its correlation matrix, in order to get rid of inter-individual differences and make the values in the connectivity matrices comparable across datasets. The freely available Brain Connectivity Toolbox (2015-01-25 version) was used for analyses of the matrices in Matlab^34^.

#### Integration of network metrics over a range of sparsity thresholds

By retaining a fixed percentage of edges, the graphs were converted into equi-sparse networks. A fixed percentage of edges has to be retained in order to have the same density between the individual networks to make them comparable. To determine systematic effects on topological organization that would be independent of the choice of a single arbitrary density threshold, we selected a range of thresholds. The lower threshold of 25% corresponded to the value at which networks for each subject and measurement remained connected, meaning there were no infinite path lengths for any node^35^. The upper threshold of 45% was the highest density with no negatively weighted edges present^27,33^. These thresholds were identical to the ones used for ketamine single injection datasets^16^. After calculating graph metrics for each threshold, we computed the area under the curve (AUC) for each network metric by averaging the metric values within the selected range of thresholds (25–45%). The AUC method is sensitive to topological alterations in psychiatric disorders, and thus has been extensively used in brain network studies^36,37^. The groups were then compared based on the AUC parameters.

#### Global and local metrics

For each network and sparsity level, we calculated five global metrics: characteristic path length (*λ*)—the average shortest path length, global clustering coefficient (*γ*)—the average of all nodal clustering coefficients, small-world index (*σ*)—the ratio of global clustering coefficient and characteristic path length, global efficiency—the average inverse shortest path length, local efficiency—the average of local efficiency values for all nodes, and five local network metrics: degree—the number of edges connecting a given node to other nodes, strength—the sum of nodal edges weights, betweenness centrality—the percentage of all shortest paths in the network containing the given node, clustering coefficient—the number of edges between the neighbors of a given node normalized to the maximum number of possible edges, local efficiency—global efficiency computed on a node’s neighborhood. To control for possible differences in the overall connectivity strength, a resampling algorithm created 100 reference random networks for each network with preserved degree and strength distributions^38^.

### Statistical analysis I. Strain-specific effects of ketamine’s repeated versus single types of administration

Since our aim was to compare the effect of ketamine’s repeated treatment to single administration, we included fMRI scans from our previous study in which the animals received only a single ketamine injection^16^. As an effect of saline was of no interest per se and if used as an additional factor would have hindered an interpretation of analysis of variance (ANOVA) results, we subtracted mean saline values from ketamine scans for each of the network properties, treating them as a reference:

Δ-Ketamine_repeated_ = Ketamine_repeated_ – mean(Saline_repeated_),

Δ-Ketamine_single_ = Ketamine_single_ – mean(Saline_single)._

Then for each delta value, we calculated a two-way ANOVA with factors “group” (NC/PC) and “type of administration” (repeated/single) (*p* < 0.05, false discovery rate (FDR) correction for number of regions *N* = 43, *q* < 0.05).

### Statistical analysis II. Strain-specific effects of repeated ketamine versus repeated saline administration

To test, whether ketamine’s effect differed from placebo (saline), when injected repeatedly, we used an ANOVA with factors “group” (NC/PC) and “treatment” (ketamine/saline) (*p* < 0.05, FDR correction, *q* < 0.05).

## Results

### Comparison of repeated to single ketamine administration

#### Global metrics

**Fig. 2:**
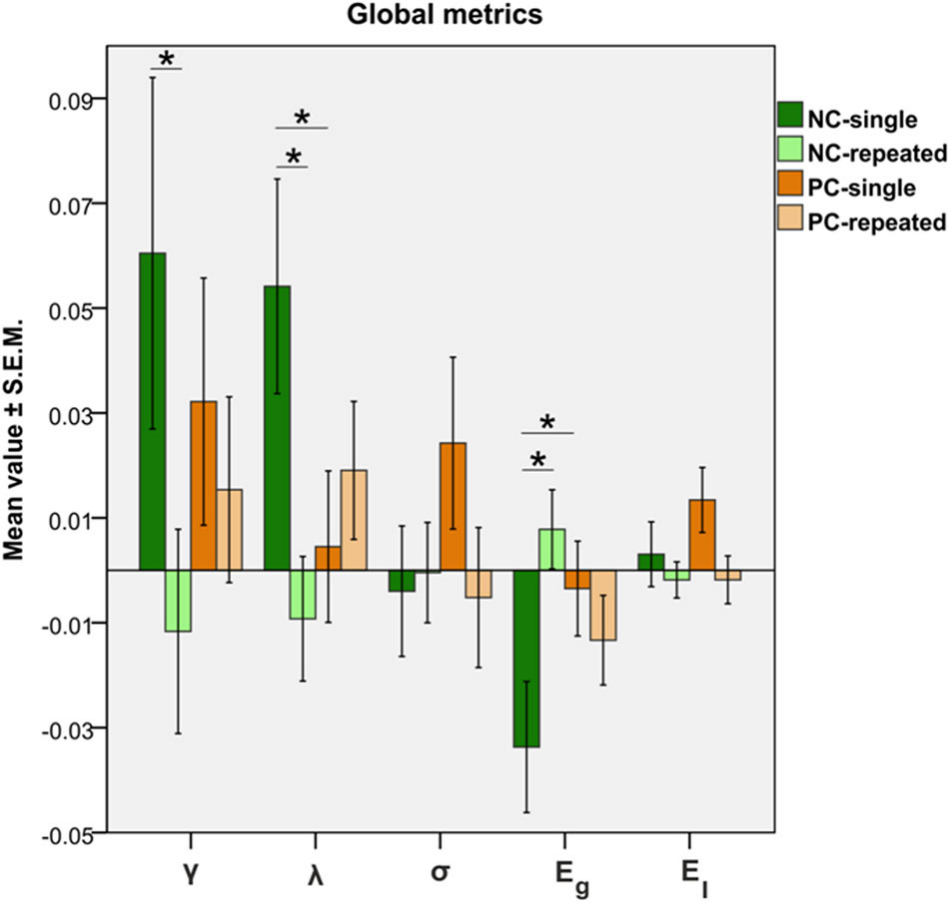
Ketamine effects on global graph analytical properties. Asterisks (*) indicate significance in the post hoc comparisons between chronic and single administration of ketamine in the NC and PC groups (*p* < 0.05). *γ* global clustering coefficient, *λ* characteristic path length, *σ* small-world index, *Eg* global efficiency, *El* local efficiency.

Repeated ketamine administration elicited a strain-specific change in characteristic path length (interaction, *F*_1,46_ = 6.5379, *p* = 0.0139) and global efficiency (interaction, *F*_1,46_ = 7.2677, *p* = 0.0098). A comparison of these parameters between the two types of administration (repeated vs. single) in the NC group revealed a reduction of the characteristic path length (*p* = 0.0043, FDR < 0.05) (lower *λ* in the NC-repeated group (light green), as compared to the NC-single (green) and no significant difference in the PC rats (orange vs. light orange in Fig. 2)) and an increase in global efficiency (*p* = 0.0029, FDR < 0.05) for repeated treatment (higher Eg in the NC-repeated group (light green), as compared to the NC-single (green) and no significant difference in the PC rats (orange vs. light orange in Fig. 2)). The reference values of each of the global metrics before subtraction and creating Δ-values are presented in Table S1.

### Local metrics

**Fig. 3:**
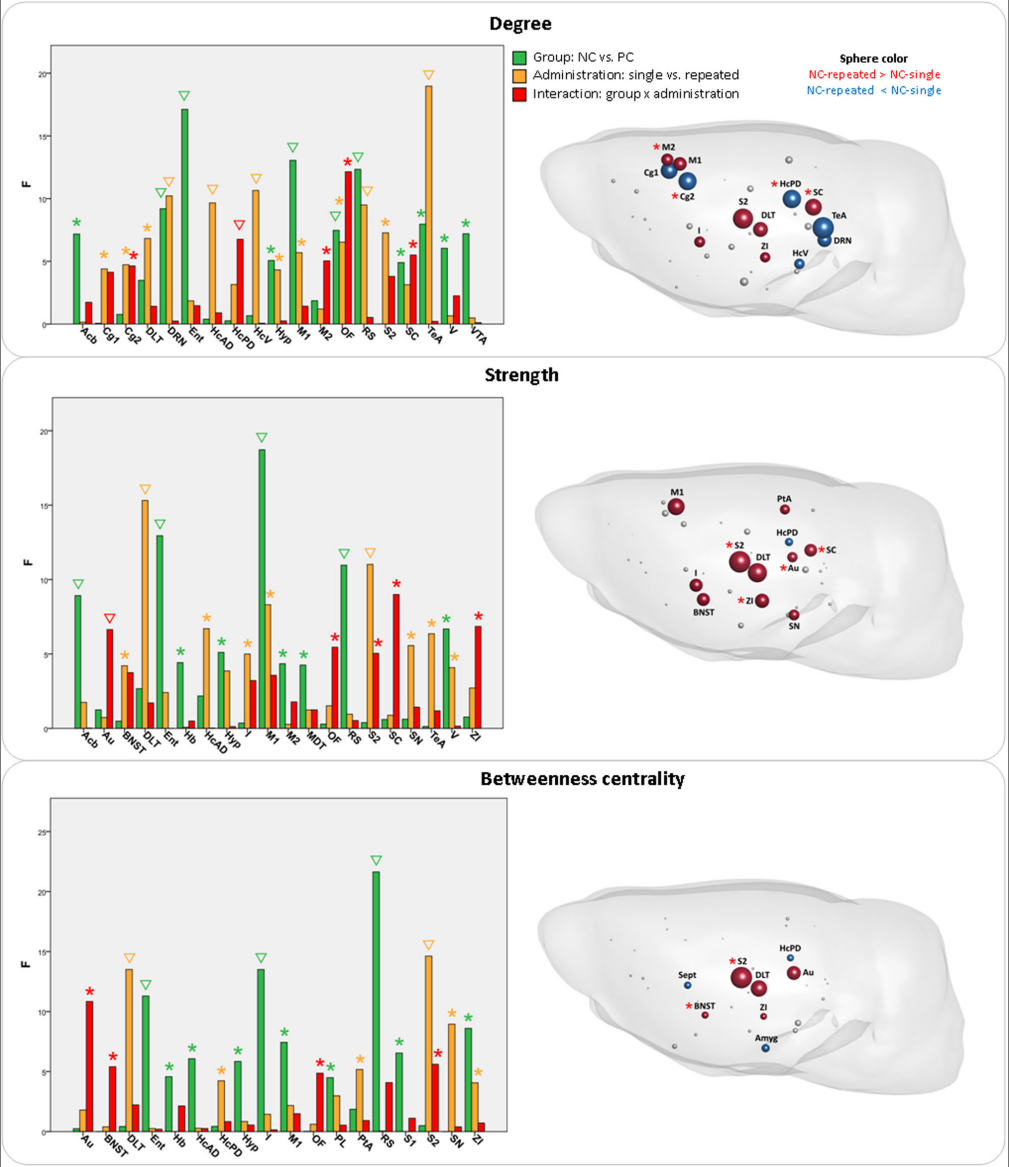
Ketamine effects on graph analytical local properties: degree, strength, and betweenness centrality. Left: The vertical bars represent *F-*statistic values from two-way ANOVA test with effects of group (green), type of ketamine administration (yellow), and interaction between group and administration (red). Asterisks (*) denote significant results (*p* < 0.05), triangles (∇) signify results surviving false discovery rate correction (correction for number of brain regions *N* = 43, *q* < 0.05). Right: Comparison between repeated and single administration of ketamine in the NC group, illustrating regions with significant effects of interaction (red asterisk) from two-way ANOVA, as shown on the left panel. The regions without asterisk were significantly different in the post hoc tests (*p* < 0.05), but had no differences for the interaction in two-way ANOVA. Several regions had significant results in interaction, but no difference between the type of administration in the NC rats in post hoc tests. Sphere size represents the –log(*p*) values from the post hoc tests, sphere color signifies the direction of the effect (red: repeated > single; blue: repeated < single). *Acb* nucleus accumbens, *Amyg* amygdala, *Au* auditory cortex, *BNST* bed nucleus of stria terminalis, *Cg1* cingulate cortex area 1, *Cg2* cingulate cortex area 2, *DLT* dorsolateral thalamus, *DRN* dorsal raphe nuclei, *Ent* entorhinal cortex, *Hb* habenula, *HcAD* antero-dorsal hippocampus, *HcPD* postero-dorsal hippocampus, *HcV* ventral hippocampus, *Hyp* hypothalamus, *I* insular cortex, *M1* primary motor cortex, *M2* secondary motor cortex, *OF* orbitofrontal cortex, *PL* prelimbic cortex, *PtA* parietal association cortex, *RS* retrosplenial cortex, *S1* primary somatosensory cortex, *S2* secondary somatosensory cortex, *SC* superior colliculus, *Sept* septal area, *SN* substantia nigra, *TeA* temporal association cortex, *V* visual cortex, *VTA* ventral tegmental area, *ZI* zona incerta.

**Fig. 4:**
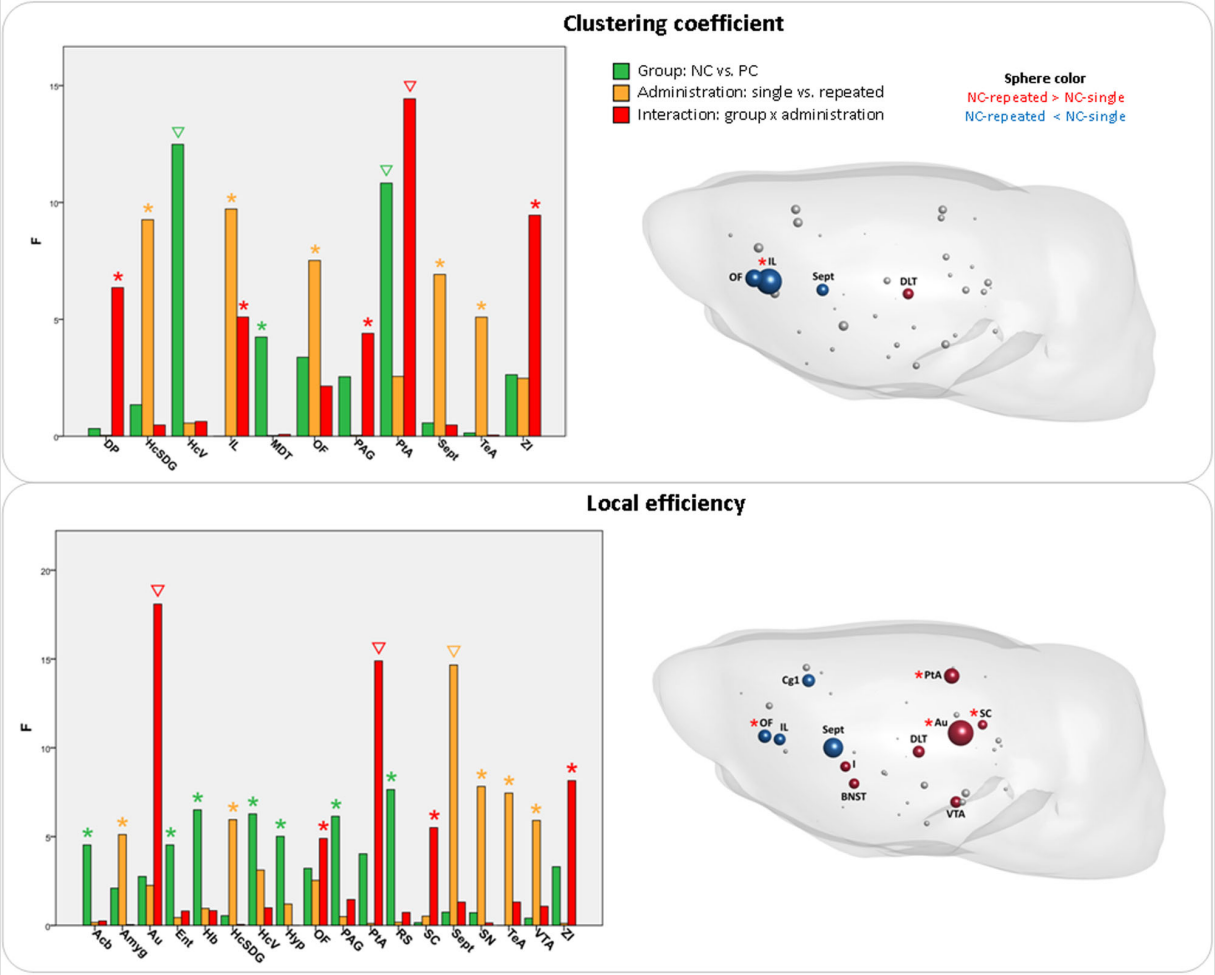
Ketamine effects on graph analytical local properties: clustering coefficient and local efficiency. See legend of the Fig. 3 for the detailed description and abbreviations of the already depicted brain regions. Abbreviations of not yet depicted brain regions: *DP* dorsal peduncular cortex, *HcSDG* subiculum and dentate gyrus parts of the hippocampus, *IL* infralimbic cortex, *MDT* midline dorsal thalamus, *PAG* periaqueductal gray.

Several graph metrics expressed interaction effects between rat strain and type of ketamine administration in the prefrontal and frontal cortical regions, such as the infralimbic (clustering, *F*_1,46_ = 5.0973, *p* = 0.0288), orbitofrontal (degree, *F*_1,46_ = 12.1477, *p* = 0.0011; strength, *F*_1,46_ = 5.4572, *p* = 0.0239; betweenness centrality, *F*_1,46_ = 4.8688, *p* = 0.0324; local efficiency, *F*_1,46_ = 4.8944, *p* = 0.0319), dorsal peduncular (clustering, *F*_1,46_ = 6.3568, *p* = 0.0152), and cingulate cortices (degree, *F*_1,46_ = 4.6352, *p* = 0.0366) (Figs. 3 and 4). The pronounced interaction effects were also present in the posterior cortical (auditory cortex: strength, *F*_1,46_ = 6.6431, *p* = 0.0132; betweenness centrality, *F*_1,46_ = 10.8406, *p* = 0.0019; local efficiency, *F*_1,46_ = 18.0898, *p* = 0.0001; parietal association cortex: clustering, *F*_1,46_ = 14.4347, *p* = 0.0004; local efficiency, *F*_1,46_ = 14.8902, *p* = 0.0004) and somato-motor areas (secondary motor cortex: degree, *F*_1,46_ = 5.0404, *p* = 0.0296; somatosensory cortex: strength, *F*_1,46_ = 5.0466, *p* = 0.0295; betweenness centrality, *F*_1,46_ = 5.6097, *p* = 0.0221).

The subcortical regions manifested interaction effects in the zona incerta (strength, *F*_1,46_ = 6.8532, *p* = 0.0119; clustering, *F*_1,46_ = 9.4532, *p* = 0.0035; local efficiency, *F*_1,46_ = 8.1497, *p* = 0.0064) and superior colliculus (degree, *F*_1,46_ = 5.5051, *p* = 0.0233; strength, *F*_1,46_ = 8.9966, *p* = 0.0044; local efficiency, *F*_1,46_ = 5.5032, *p* = 0.0233).

Most of these interaction effects arose from the differences between repeated and single administration within the NC group, as demonstrated by the post hoc pairwise comparisons (Fig. S1). Importantly, the direction of changes after repeated ketamine in the prefrontal areas qualitatively corresponded to the direction of the local topological metrics in the NC strain baseline pre-ketamine state, as compared to the PC strain, in our previous study^16^, with orbitofrontal, infralimbic, and cingulate cortices displaying reduced degree, clustering, and efficiency (Figs. 3, 4, and S1 and Table 1). On the contrary, the somato-motor, parietal association, and auditory cortices had enhanced values of almost all graph parameters for repeated treatment (Figs. 3, 4, and S1 and Table 1). Similarly, the zona incerta and superior colliculus exhibited increased values of degree, strength, betweenness centrality, or local efficiency (Figs. 3, 4, and S1 and Table 1).

**Table 1:** The post hoc comparison of repeated ketamine vs. single ketamine in rats bred for negative (NC) cognitive state for local graph analytical metrics (increase ↑ or decrease ↓). Triangle (∇) signifies values surviving FDR correction (*q* < 0.05). See legends of Figs. 3 and 4 for the abbreviations of brain regions.

Brain regions	*p* Value				
	Degree	Strength	Betweenness centrality	Clustering coefficient	Local efficiency
Amyg			↓ 0.0230		
Au		↑ 0.0169	↑ 0.0016^∇^		↑ 0.0001^∇^
BNST		↑ 0.0060^∇^	↑ 0.0384		↑ 0.0244
Cg1	↓ 0.0046^∇^				↓ 0.0097
Cg2	↓ 0.0031^∇^				
DLT	↑ 0.0086^∇^	↑ 0.0005^∇^	↑ 0.0005^∇^	↑ 0.0307	↑ 0.0134
DRN	↓ 0.0107^∇^				
HcPD	↓ 0.0028^∇^	↓ 0.0390	↓ 0.0375		
HcV	↓ 0.0349				
I	↑ 0.0307	↑ 0.0056^∇^			↑ 0.0237
IL				↓ 0.0003^∇^	↓ 0.0140
M1	↑ 0.0130^∇^	↑ 0.0012^∇^			
M2	↑ 0.0200				
OF				↓ 0.0039^∇^	↓ 0.0086^∇^
PtA		↑ 0.0191			↑ 0.0040^∇^
S2	↑ 0.0016^∇^	↑ 0.0002^∇^	↑ 0.0001^∇^		
SC	↑ 0.0047^∇^	↑ 0.0066^∇^			↑ 0.0318
Sept			↓ 0.0333	↓ 0.0206	↓ 0.0008^∇^
SN		↑ 0.0136			
TeA	↓ 0.0011^∇^				
VTA					↑ 0.0159
ZI	↑ 0.0377^∇^	↑ 0.0035^∇^	↑ 0.0445		

### Comparison of repeated ketamine to saline

Global metrics displayed no differences between the groups or the treatment. For the local metrics repeated ketamine, as compared to saline, had a very modest effect. Strain-specific effects appeared only in a few of the cortical regions, such as the retrosplenial (degree, *F*_1,43_ = 4.54, *p* = 0.0388), entorhinal (degree, *F*_1,43_ = 5.98, *p* = 0.0186), dorsal peduncular (clustering coefficient, *F*_1,43_ = 4.52, *p* = 0.0393), auditory (local efficiency, *F*_1,43_ = 11.14, *p* = 0.0018), and parietal association cortices (clustering coefficient, *F*_1,43_ = 8.98, *p* = 0.0045; local efficiency, *F*_1,43_ = 8.99, *p* = 0.0045) (Table S2).

The post hoc pairwise comparisons demonstrated some of these changes to result from effects of ketamine either within the NC group, for example, in the entorhinal cortex (degree, NC-ketamine < NC-saline, *p* = 0.0408) and auditory cortex (local efficiency, NC-ketamine > NC-saline, *p* = 0.0001, survived FDR), or between NC and PC groups, as in dorsal peduncular cortex (clustering coefficient, NC-ketamine < PC-ketamine, *p* = 0.0171) and parietal association cortex (local efficiency, NC-ketamine > PC-ketamine, *p* = 0.0391).

## Discussion

Repeated ketamine, as compared to single injection, evoked a strain-specific brain network randomization effect, expressed as enhanced global efficiency and reduced path length in the depressive-like NC rats. This effect strikingly resembles the depressive-like strain’s pre-ketamine baseline network characteristics^16^ and those of depressed patients^37,39,40^. Similarly at the nodal level, the strain-specific reduced prefrontal connectivity in the NC rats mirrored the depressive pattern. Finally, the scarcity of differences between the repeated ketamine and placebo (saline) stresses out an inefficacy of repeated treatment at least at the network level.

### Strain-specific effects of repeated vs. single ketamine

Repeated application of ketamine induced prefrontal topological changes in the NC rats similar to their baseline state^16^ and common network phenotypes of clinical depressive state^41–43^, expressed as reduced degree for the cingulate cortex and reduced local efficiency for the orbitofrontal cortex. Conversely, the somato-motor cortical regions displayed strain-specific increased degree, strength and betweenness centrality. As degree and strength signify number and weights of connections, this change may rather reflect an increased dendritic spine formation in the somatosensory cortex, reported in mice treated for 5 days with 10 mg/kg ketamine^44^.

Several other areas involved in sensory and motor functions manifested strain-specific effects: these included the auditory cortex and superior colliculus mediating auditory and visual perception, the parietal association cortex providing sensory-to-motor transformation^45^, and the zona incerta participating in limbic-motor integration^46^. While it is difficult to speculate about the functional meaning of these findings, these effects might possibly explain the long-lasting perceptual and motor-related cumulative side effects. For example, an increase in strength, betweenness centrality, and local efficiency in the auditory cortex resembles a general strain-independent acute effect of ketamine in both NC and PC rats^16^, as well as in healthy Sprague–Dawley rats from our previous study^27^. This increase might reflect a lasting effect on auditory perception, as chronic ketamine elicits reductions in several parameters of the auditory event-related potentials in humans^47^ and in rodents produces chronic reductions in auditory-evoked potentials^48^. In parallel, the visual function could also be affected, as repeated ketamine enhanced degree, strength, and efficiency of the superior colliculus, a midbrain structure receiving connections from the retinal ganglion cells and involved in the generation of saccades and in visual orienting behavior toward novel, unexpected or salient stimuli^49^. This function is directly linked to distractibility, an inability to sustain attention^50,51^, manifested in clinical depression^52^ and induced by ketamine^53^. Collicular lesions reduce distractibility^54^, whereas hyper-responsive superior colliculus could enhance this condition^51^. Thus, in addition to the modulation of the visual perception^47,55^, repeated ketamine’s effect on collicular topological properties might underlie attentional deficits.

### Effects of repeated ketamine vs. placebo

It is important to note that, in contrast to most of the investigations on repeated ketamine, our study included a placebo (control) group. Only five recent human studies included a control group^9,55–58^, and, strikingly, they showed contradictory results. Ketamine either maintained antidepressant efficacy^9,56,57^ or failed to outperform placebo^58^ and was ineffective^55^, additionally causing side effects^55,58^. The study of Canuso et al. ^56^ demonstrated that although the depressive symptoms diminish after ketamine at all time points, the suicidal ideation reduces only at 4 h, and displays no improvement at 24 h or at end-point day 25. The reasons for these mixed outcomes are unclear and might include higher treatment resistance levels^58^, chronic history of depression^58^, ineffective dosing, and possible tolerance-like phenomena^55,58,59^. For example, Sprague–Dawley rats develop tolerance to repeated administration of anesthetic doses of ketamine, possibly arising as a result of receptor desensitization, reduction of receptor density, or increased induction of metabolizing enzymes^60^. Also, a recent animal study comparing the effects of a single and repeated treatment of ketamine demonstrated the lack of antidepressant-like effectiveness of long-term administration^61^, hypothesizing to result from the detrimental effects of the parvalbumin interneurons loss in the prefrontal cortex after repeated treatment^62^ and/or possible metabolic side effects.

On top of that, most studies, except a few^56,58^, excluded patients with high suicide risk. The study demonstrating an antidepressant efficacy of repeated ketamine^9^ had exclusion criteria of suicidal ideation and history of non-response to ketamine. On the contrary, the study including patients with both severe TRD and current chronic suicidal ideation showed a failure of ketamine to surpass placebo^58^. The absence of difference to placebo may reflect the fact that severely depressed patients have decreased responsiveness to placebo, rather than increased responsiveness to medication^63^. In agreement with this suggestion, our additional analysis comparing repeated ketamine to saline detected very few strain-specific differences. This is consistent with the results of the human placebo-controlled studies^55,58^ and might similarly result from an inability of repeated ketamine to surpass placebo effects in this TRD model. Out of 18 assessed rodent models of depression, the genetic model used in the current study is one of the most robust and reliable^18^. It models depression resistant to antidepressant (desipramine) and electroconvulsive therapy^64^. Ketamine’s single injection is effective in this model^23,65,66^; however, repeated treatment was tested only in the chronic unpredictable stress and chronic mild stress models of depression^13–15^, and it remains to be tested in this cognitive model.

The possibility of a differential neural mechanism underlying ketamine’s antidepressant effect, which would not be reflected in network topology, also cannot be excluded. This might be represented by neuroplastic changes, levels of brain-derived neurotrophic factor and corticosterone, gene expression, and molecular pathway changes, among many possible reasons. Therefore, we would like to stress that we show a lack of long-term efficacy of repeated treatment only from a network point of view.

## Conclusions

The strain-specific effect of repeated ketamine treatment on brain topology resembled the depressive-like imaging phenotype and showed an effect highly dissimilar to single application. Several of the affected regions belonging to the auditory and visual circuitry hint at possible cumulative side effects at the sensory level. The scarcity of differences between the repeated ketamine and placebo (saline) indicates an inefficacy of repeated treatment, at least on the network topology, and is supported by the lack of antidepressant efficacy of repeated treatment reported in recent placebo-controlled studies. However, differential mechanisms for an antidepressant effect of repeated ketamine not reflected in network topology cannot be excluded. Further behavioral validation and correlation with imaging data would be necessary to ascertain the functional significance of our findings. Nevertheless, this empirical evidence is an advance in understanding the underlying neurobiological basis of both types of treatment.

## Supplementary information

Supplementary
