# Supplementary material for: The influence of ketamine’s repeated treatment on brain topology does not suggest an antidepressant efficacy
Source: Transl Psychiatry. 2020 Feb 4;10:56. doi: 10.1038/s41398-020-0727-8 (PMC7026038; doi:10.1038/s41398-020-0727-8)
Supplement: Supplementary file 1 — Supplementary [file 41398_2020_727_MOESM1_ESM.doc]

**Supplemental Material for:**

N. Gass, R. Becker, J. Reinwald, A. Cosa-Linan, M. Sack, W. Weber-Fahr, B. Vollmayr, A. Sartorius.

**The influence of ketamine’s repeated treatment on brain circuits does not support antidepressant efficacy.**

**Experimental procedures**

**Test for escape behavior.** Since in later (more than 20) generations an escape deficit occured even in absence of prior inescapable shocks, the behavioral test was conducted without uncontrollable shocks to minimize animal suffering (1). The chambers had dimensions of 48.5 x 30 x 21.5 cm with the floor built of steel rods (6 mm in diameter, 20 mm apart) and had a 35x35 mm lever positioned on one side. The experiment was controlled by computer. The boxes, shock generator and controlling program were obtained from TSE, Bad Homburg, Germany. The test consisted of 10 trials of 0.8 mA current, each lasting 60 s with inter-trial time 24 s, if a rat did not stop the current by pressing the lever. The latency to stop a trial was recorded.

**Results**

**
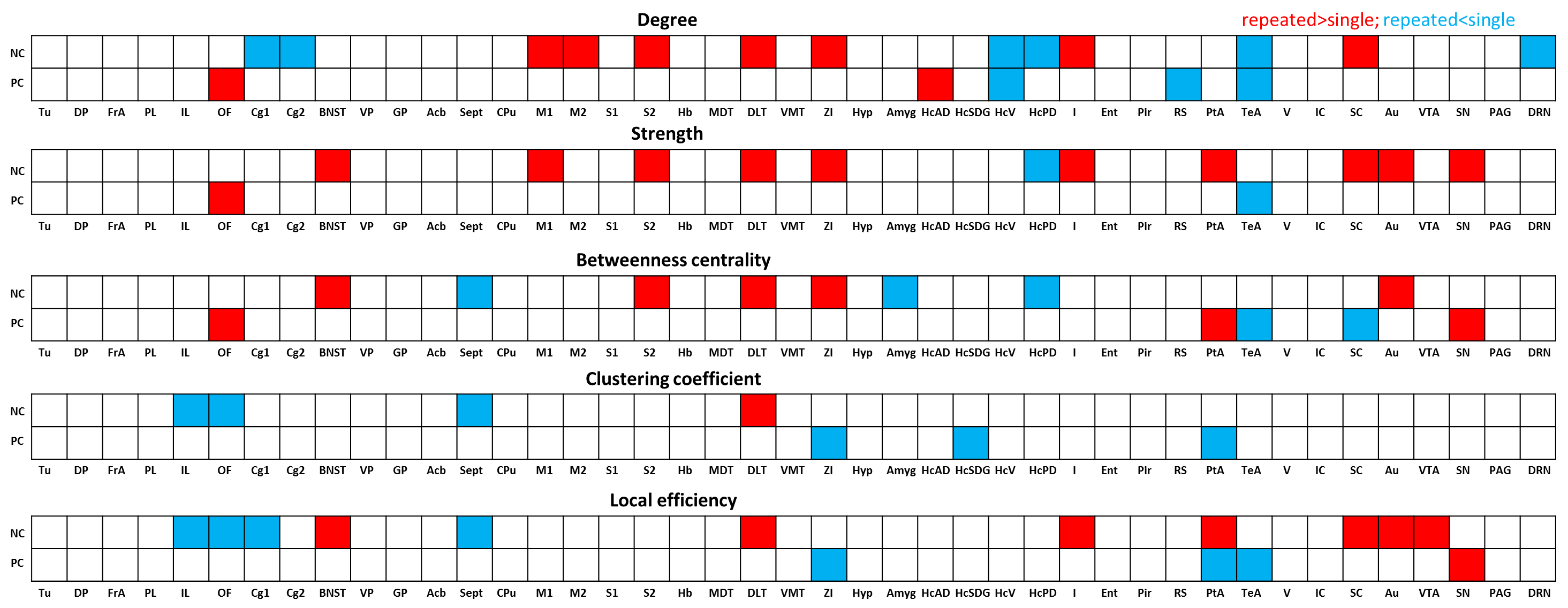
**

**Figure S1.** **Differences in the graph analytical local properties between repeated and single ketamine treatment within the NC and PC groups.** Blue color depicts values lower for the repeated ketamine, as compared to the single type of administration, red color – higher values for the repeated ketamine. Brain regions: *Acb* - nucleus accumbens; *Amyg* – amygdala; *Au* -auditory cortex; *BNST* - bed nucleus of stria terminalis; *CPu* - caudate-putamen; *Cg1* - cingulate cortex, area 1; *Cg2* - cingulate cortex, area 2; *DLT* - dorsolateral thalamus; *DP* - dorsal peduncular cortex; *DRN* - dorsal raphe nuclei; *Ent* - entorhinal cortex; *FrA* - frontal association cortex; *GP* – globus pallidus; *Hb* - habenula; *HcAD* - hippocampus, anterodorsal; *HcPD* - hippocampus, posterodorsal; *HcSDG* - hippocampus, subiculum and dentate gyrus; *HcV* – hippocampus, ventral; *Hyp* - hypothalamus; *I* - insular cortex; *IC* - inferior colliculus; *IL* - infralimbic cortex; *M1* - primary motor cortex; *M2* - secondary motor cortex; *MDT* - midline dorsal thalamus; *OF* - orbitofrontal cortex; *PAG* - periaqueductal gray; *Pir* - piriform cortex; *PL* - prelimbic cortex; *PtA* - parietal association cortex; *RS* - retrosplenial cortex; *S1* - primary somatosensory cortex; *S2* - secondary somatosensory cortex; *SC* - superior colliculus; *Sept* - septum; *SN* – substantia nigra; *TeA* - temporal association cortex; *Tu* – olfactory tubercle; *V* - visual cortex; *VMT* - ventromedial thalamus; *VP* - ventral pallidum; *VTA* - ventral tegmental area; *ZI* - zona incerta.

| **Groups** | **Clustering coefficient γ** | | **Small-worldness index σ** | | **Characteristic path length λ** | | **Local efficiency**  **E_l_** | | **Global efficiency**  **E_g_** | |
| --- | --- | --- | --- | --- | --- | --- | --- | --- | --- | --- |
|  | **ketamine** | **saline** | **ketamine** | **saline** | **ketamine** | **saline** | **ketamine** | **saline** | **ketamine** | **saline** |
| **NC-repeated** | 1.2398 | 1.2515 | 1.1765 | 1.1769 | 1.0536 | 1.0628 | 1.0597 | 1.0616 | 0.9606 | 0.9528 |
| **PC-repeated** | 1.2834 | 1.2681 | 1.1895 | 1.1947 | 1.0795 | 1.0605 | 1.0683 | 1.0701 | 0.9405 | 0.9538 |
| **NC-single** | 1.2900 | 1.2295 | 1.1764 | 1.1804 | 1.0956 | 1.0415 | 1.0662 | 1.0632 | 0.9324 | 0.9661 |
| **PC-single** | 1.2767 | 1.2445 | 1.1726 | 1.1483 | 1.0890 | 1.0845 | 1.0595 | 1.0461 | 0.9356 | 0.9391 |

**Table S1.** The reference values of each of the global metrics before subtraction and creation of the Δ-values.

| **Brain regions** | **F-value** | | | | |
| --- | --- | --- | --- | --- | --- |
|  | **Degree** | **Strength** | **Betweenness**  **centrality** | **Clustering**  **coefficient** | **Local efficiency** |
| **Acb** |  |  | F(Group)_1,43_=4.32, p=0.0438 |  |  |
| **Au** |  |  |  | F(Group)_1,43_=14.33^∇^, p=0.0005 | F(Group)_1,43_=11.72, p=0.0014  F(Treatment)_1,43_=5.30, p=0.0262  F(Interaction)_1,43_=11.14, p=0.0018 |
| **Cg1** | F(Group)_1,43_=4.91, p=0.0320 |  |  |  |  |
| **CPu** |  |  |  | F(Group)_1,43_=4.62, p=0.0373 |  |
| **DP** |  |  |  | F(Interaction)_1,43_=4.52, p=0.0393 |  |
| **DRN** | F(Treatment)_1,43_=7.28, p=0.0099 |  |  |  |  |
| **Ent** | F(Interaction)_1,43_=5.98, p=0.0186 |  |  |  |  |
| **HcSDG** |  | F(Group)_1,43_=5.02, p=0.0303 |  |  |  |
| **HcV** | F(Treatment)_1,43_=4.38, p=0.0422 | F(Group)_1,43_=5.62, p=0.0223 |  |  |  |
| **M2** |  |  | F(Treatment)_1,43_=5.25, p=0.0269 |  |  |
| **PtA** |  |  |  | F(Treatment)_1,43_=5.10, p=0.0291  F(Interaction)_1,43_=8.98, p=0.0045 | F(Treatment)_1,43_=5.05, p=0.0298  F(Interaction)_1,43_=8.99, p=0.0045 |
| **RS** | F(Treatment)_1,43_=5.37, p=0.0253  F(Interaction)_1,43_=4.54, p=0.0388 |  |  |  |  |
| **S2** |  | F(Treatment)_1,43_=4.75, p=0.0349 | F(Treatment)_1,43_=9.67, p=0.0033 |  |  |
| **VP** |  |  |  |  | F(Treatment)_1,43_=5.55, p=0.0231 |

**Table S2.** **F and p-values for local graph analytical metrics of individual brain regions from ANOVA comparison of repeated ketamine versus repeated saline administration.** Triangle (^∇^) signifies values which survived FDR correction (corrected for number of brain regions N=43, q<0.05). Brain regions: *Acb* - nucleus accumbens; *Au* -auditory cortex; *Cg1* - cingulate cortex, area 1; *CPu* - caudate-putamen; *DP* - dorsal peduncular cortex; *DRN* - dorsal raphe nuclei; *Ent* - entorhinal cortex; *HcSDG* - hippocampus, subiculum and dentate gyrus; *HcV* – hippocampus, ventral; *M2* - secondary motor cortex; *PtA* - parietal association cortex; *RS* - retrosplenial cortex; *S2* - secondary somatosensory cortex; *VP* - ventral pallidum.

**References**

(1) Richter SH, Sartorius A, Gass P, Vollmayr B. A matter of timing: harm reduction in learned helplessness*. Behav Brain Func*t 2014; 10: 41-9081-10-41.
